# MEG2 is regulated by miR-181a-5p and functions as a tumour suppressor gene to suppress the proliferation and migration of gastric cancer cells

**DOI:** 10.1186/s12943-017-0695-7

**Published:** 2017-07-26

**Authors:** Zhijian Liu, Feng Sun, Yeting Hong, Yanqing Liu, Min Fen, Kai Yin, Xiaolong Ge, Feng Wang, Xi Chen, Wenxian Guan

**Affiliations:** 1 Department of Gastrointestinal Surgery, Nanjing Drum Tower Hospital, The Affiliated Hospital of Nanjing University Medical School, 321 Zhongshan Road, Nanjing, Jiangsu 210008 China; 2 State Key Laboratory of Pharmaceutical Biotechnology, Jiangsu Engineering Research Center for MicroRNA Biology and Biotechnology, NJU Advanced Institute for Life Sciences (NAILS), School of Life Sciences, Nanjing University, 163 Xianlin Road, Nanjing, Jiangsu 210046 China; 3 Department of General Surgery, Sir Run Run Shaw Hospital, School of Medicine, Zhejiang University, East Qingchun Road, Hangzhou, 310016 China

**Keywords:** Gastric cancer, Protein-tyrosine phosphatase MEG2, miR-181a-5p, microRNA

## Abstract

**Background:**

Protein-tyrosine phosphatase MEG2 (MEG2) is a classic tyrosine-specific protein tyrosine phosphatase (PTP). It has been reported that MEG2 participates in the carcinogenesis of the breast and liver. However, functions of MEG2 in gastric cancer remain poorly understood.

**Methods:**

We examined the expression of MEG2 protein by western blotting and that of miR-181a-5p by qRT-PCR. We used bioinformatic analyses to search for miRNAs that potentially target MEG2. We performed a luciferase reporter assay to investigate the interaction between miR-181a-5p and MEG2. In addition, we assessed the effects of MEG2 and miR-181a-5p on gastric cancer cells in vitro and in vivo.

**Results:**

We found that MEG2 is downregulated in human gastric cancer and that miR-181a-5p is predicted to be a potential regulator of MEG2. We also observed that expression of MEG2 is reversely correlated with that of miR-181a-5p in gastric cancer. Moreover, we observed that MEG2 regulation by miR-181a-5p significantly suppresses the proliferation and migration of gastric cancer cells in vitro and decelerates tumour growth in vivo.

**Conclusions:**

Our results revealed that MEG2 is a tumour suppressor gene and negatively regulated by miR-181a-5p in gastric cancer.

**Electronic supplementary material:**

The online version of this article (doi:10.1186/s12943-017-0695-7) contains supplementary material, which is available to authorized users.

## Background

Gastric cancer (GC) is one of the most common cancer-related causes of death throughout the world [1]. Approximately 1000, 000 new cases of GC occur every year, and more than 50% of these cases occur in Eastern Asia, especially in China. Since many GC cases have a high rate of lymph node metastasis and are diagnosed at an advanced stage, they result in one of the highest mortality rates among all types of cancer, with the 5-year survival rate being as low as 40% in China [2, 3]. Thus, it is urgent to understand the molecular mechanisms of GC to help develop new therapeutic strategies.

Protein tyrosine phosphatases (PTPs) play vital roles in determining the levels of cellular tyrosine phosphorylation and in the regulation of many important signalling pathways, including JAK/STAT3 and EGFR/RAS/MAPK [4, 5]. For instance, as a classical PTP, DEP1 inhibits cancer cell proliferation by directly dephosphorylating of EGFR (epidermal growth factor receptor) in the cytoplasm [6]. TC45 (the nuclear isoform of TC-PTP), another member of PTPs family, can dephosphorylate STAT3 (signal transducer and activator of transcription 3) in the nucleus mediated by GdX (Ubiquitin-like protein 4A) [7]. Protein-tyrosine phosphatase MEG2 (MEG2) belongs to the family of PTPs [8]. MEG2 was reported to regulate erythroid cells expansion, controls embryonic development, and modulates secretory vesicle fusion [9–11]. Other functional studies showed that MEG2 regulates insulin signaling pathway and beta cell growth through directly dephosphorylating insulin receptor in diabetes [12]. Recent studies also reported that MEG2 is a negative regulator in breast cancer and hepatocellular carcinoma by promoting the dephosphorylation of EGFR, HER-2 and STAT3 [13, 14]. However, it is unknown whether MEG2 also acts as a tumour suppressor gene in GC.

MicroRNAs (miRNAs) are a family of small, non-coding RNAs that are 19 ~ 22 nucleotides in length. They play a significant role in the regulation of gene expression at post-transcriptional levels [15, 16]. Functionally, miRNAs bind to complementary sites in the 3′-untranslated regions (3′-UTRs) of target mRNAs, resulting in mRNA translational suppression and/or degradation. Thus, the expression of the target gene is suppressed [16–18]. Dysfunction of miRNAs is implicated in the tumourigenesis of various cancers, including GC, and these miRNAs can act as tumour suppressor miRNAs or oncomiRs [19–21].miR-181a-5p is widely known to be associated with the development and differentiation of blood vascular endothelial cells [22] and lymphocytes [23]. However, miR-181a-5p also plays a vital role in many cancers, including multiple myeloma [24], breast cancer [25], leukaemia [26], and hepatocellular carcinoma [27]. Recent studies have found that miR-181a-5p is upregulated in GC specimens [28, 29]. However, its precise function and mechanism in this type of cancer has not been systematically studied and needs further researches.

In the present study, we found that the MEG2 level is downregulated and is reversely correlated with the expression of miR-181a-5p in GC. We thus predicted by bioinformatics that miR-181a-5p is a potential regulator of MEG2 and further determined that MEG2 is directly inhibited by miR-181a-5p in vitro. Consequently, we demonstrated that MEG2, regulated by miR-181a-5p, suppresses the proliferation and migration of gastric cancer cells in vitro and decelerates gastric tumour growth in vivo.

## Methods

### Human specimens and cells

Paired human gastric cancer (GC) and gastric normal (GN) tissue samples were provided by Nanjing Multi-center Biobank, Biobank of Nanjing Drum Tower Hospital, the Affiliated Hospital of Nanjing University Medical School, with the consent of every donor. The samples were normalized for ethnicity. The samples were frozen at the time of surgery in liquid nitrogen and then stored at −80 °C. The clinical characteristics of the patients are listed in Additional file 1: Table S1. The human gastric cancer cell lines (MGC803, SGC7901, MKN-45, HGC-27 and BGC-823) and the human gastric epithelial cell line (GES-1) were purchased from the Shanghai Institute of Cell Biology, Chinese Academy of Sciences (Shanghai, China). Both cell lines were maintained in RPMI 1640 medium supplemented with 10% foetal bovine serum (FBS, Gibco, Carlsbad, CA, USA) in a humidified atmosphere with 5% CO_2_.

### RNA isolation and quantitative RT-PCR

Total RNA was extracted from maintained cells or human tissue samples using TRIzol Reagent (Sigma, St. Louis, MO, USA), according to manufacturer’s instructions. Assays to quantify miRNAs were performed using TaqMan miRNA probes (Applied Biosystems, Foster City, CA, USA). One microgram of total RNA was briefly reverse transcribed into cDNA using the AMV reverse transcriptase (TaKaRa, Dalian, China) and a stem-loop RT primer (Applied Biosystems). The reactions were incubated at 16 °C for 30 min, followed by 42 °C for 30 min, and 85 °C for 5 min. Real-time PCR was performed in 96-well plates on a 7500 Sequence Detection System (Applied Biosystems) using a TaqMan PCR kit. The reaction conditions were as follows: 95 °C for 10 min and 40 cycles of 95 °C for 15 s and 60 °C for 1 min. The cycle threshold (CT) data were determined using fixed threshold settings, and the mean CT was determined by the triplicate PCRs. The relative expression levels of miRNAs in the cell lines and tissue samples were calculated with the eq. 2-△△CT in which △△C_T_ = (C_T miR-181a-5p_ - C_T U6_)_target_ - (C_T miR-181a-5p_ - C_T U6_)_control_.

To quantify GAPDH and MEG2 mRNA, 1 μg of total RNA was reverse transcribed to cDNA using AMV reverse transcriptase (TaKaRa) and Oligo(dT)18 primers (TaKaRa). The reactions were incubated at 42 °C for 60 min and 85 °C for 5 min. Then, real-time PCR was performed using specific primers and SYBR Green dye (Invitrogen). The primer sequences were as follows: GAPDH (sense): CGAGCCACATCGCTCAGACA and GAPDH (antisense): GTGGTGAAGACGCCAGTGGA; MEG2 (sense): CCTGCCTTAGACTGGGACT and MEG2 (antisense): TTCGCTTTGTTAGCTTCACT. The reaction conditions were as follows: 95 °C for 5 min and 40 cycles of 95 °C for 30 s, 55 °C for 30 s and 72 °C for 30 s. Upon reaction completion, the CT values were determined by setting a fixed threshold. The relative amounts of MEG2 mRNAs were normalized to GAPDH as described above.

### Protein extraction and western blotting

The cells and specimens were lysed in RIPA lysis buffer (Beyotime, Shanghai, China) freshly supplemented with 1% PMSF and incubated on ice for 30 min before being centrifuged for 15 min (12,000 r/m, 4 °C). The supernatant was collected after centrifugation, and the concentration of protein was calculated with a BCA protein assay kit (Thermo Scientific, Rockford, IL, USA). The protein levels of the cell or tissue extracts were semi-quantified by western blotting. The MEG2 expression levels of tissue extracts were detected with the immobilon western HRP Substrate (WBKLS0050, Millipore, Billerica, MA, USA). The antibodies used were anti-MEG2 (MAB2668, R&D Systems Inc., Minneapolis, MN, USA), anti-GAPDH (sc-47,724, Santa Cruz, Dallas, TX, USA).

### Plasmid construction and siRNA interference assay

A mammalian expression plasmid containing the full-length open reading frame (ORF) of the human MEG2 gene without the miR-181a-5p-responsive 3′-UTR was purchased from GeneCopoeia (Germantown, MD, USA). An empty plasmid served as the negative control. The human MEG2 siRNA was purchased from RiboBio (Guangzhou, China), and scrambled siRNA was used as a negative control. The MEG2 siRNA or overexpression plasmid was transfected into MGC803 cells using Lipofectamine 3000 (Invitrogen) as per manufacturer’s instructions. Total RNA and protein were collected 48 h after transfection. The MEG2 mRNA and protein expression levels were assessed by qRT-PCR and western blotting, respectively.

### Cell proliferation assay

Cell Counting Kit- 8 (CCK-8) is a colorimetric method to measure the quantity of living cells in cell proliferation and viability assays. MGC803 cells were proliferated using CCK-8 assays (Dojindo). MGC803 cells were seeded in 96-well plates at a density of 3 × 10^3^ cells per well at 6 h after transfection and incubated in RPMI 1640 medium supplemented with 2% FBS. At 12, 24, 36, 48 and 60 h after transfection, the absorbance value of each test well was measured at a wavelength of 450 nm, as described by the manufacturer.

### Cell migration assay

The cell migration ability was detected using Millipore 24-well Millicell (Millipore) plates with polycarbonate membranes containing 8-μm pores. The bottom surface of the membranes was coated with 0.1% gelatin. The cells were harvested 24 h after transfection, suspended in FBS-free RPMI-1640 culture medium, and then added to the upper chamber (6 × 10^3^ cells/well). Simultaneously, 0.5 mL of RPMI-1640 with 20% FBS was added to the lower compartment. The Transwell-containing plates were incubated in a humidified atmosphere with 5% CO_2_. After 24 h, the cells that had traversed through the membrane were fixed for 15 min in 4% paraformaldehyde. The membrane was washed with distilled water several times. Then, cells were stained for 15 min with 0.1% crystal violet in methanol. Finally, the bottom surfaces of the filter membranes with the migrant cells were imaged by photomicroscopy (BX51 Olympus, Japan). The cells were quantified blindly.

### miR-181a-5p overexpression and knockdown

Overexpression of miR-181a-5p was obtained by transfecting GC cells with a miR-181a-5p mimic (a synthetic RNA oligonucleotide duplex mimicking the miRNA precursor). miR-181a-5p knockdown was obtained by transfecting an miRNA inhibitor (a chemically modified single-stranded antisense oligonucleotide designed to specifically target the mature miRNA). Synthetic pre-miR-181a-5p, anti-miR-181a-5p, pre-miR-control and anti-miR-control (the scrambled negative control RNAs) were purchased from RiboBio (Guangzhou, China). MGC803 and SGC7901 cells were transfected with Lipofectamine 3000 (Invitrogen) using Opti-MEM (Gibco, Carlsbad, CA, USA), according to the manufacturer’s instructions. Pre-miR-181a-5p and pre-miR-control were used in equal doses. For the miRNA knockdown, equal amounts of anti-miR-181a-5p or anti-miR-control were used in each well. After 6 h, the medium was changed to RPMI 1640 supplemented with 2% FBS. At 24 h after transfection, the cells were collected and subjected to analysis by quantitative RT-PCR and western blotting.

### Luciferase reporter assay

To construct a luciferase reporter containing the MEG2 3′-UTR with a predicted miR-181a-5p binding site, we amplified a 1608-bp MEG2 3′-UTR region using genomic DNA as a template. The following PCR primers were used: MEG2–3′-UTR: 5′-GGACTAGTCTCTCCTACGAACCTCCTAC-3′ (forward primer) and MEG2–3′-UTR: 5′-CGACGCGTCTGTATCACTGTAAGATATTG-3′ (reverse primer). The amplified fragment was inserted into the pMIR-Report plasmid (Ambion, Austin, TX, USA). We also constructed an equivalent reporter plasmid that carried the mutant MEG2 3′-UTR region. For the luciferase reporter assays, MGC803 cells were cultured in 24-well plates and transfected with pre-miR-181a-5p, pre-miR-control, anti-miR-181a-5p or anti-miR-control in equal doses; 0.3 μg of firefly luciferase reporter plasmid; and 0.15 μg of a β-galactosidase expression vector (Ambion, Austin, TX, USA) using Lipofectamine 3000 (Invitrogen). The β-gal expression vector was used as a transfection control. Cells were assayed using luciferase assay kits (Promega, Madison, WI, USA) 24 h after transfection.

### Establishment of gastric cancer xenografts model

Five-week-old female mice (nu/nu) with severe combined immunodeficiency (SCID) were purchased from the Model Animal Research Center of Nanjing University (Nanjing, China) and maintained under specific pathogen-free conditions at Nanjing University. We established a gastric cancer cell line with stable overexpression of miR-181a-5p. miR-181a-5p overexpression lentivirus and control lentivirus were purchased from GenePharma (Shanghai, China). The detailed construct of miR-181a-5p overexpression lentiviral plasmid was presented in Additional file 2: Figure S1a. Puromycin was purchased from Sigma-Aldrich (St. Louis, USA) and added into the cells to select the stably infected MGC803 cells after 3 days of infection. After day 7, the representative fluorescence image of stably infected MGC803 cells was showed in Additional file 2: Figure S1b. The mice were subcutaneously injected under the left forelimb with MGC803 cells infected with control lentivirus, miR-181a-5p overexpression lentivirus, MEG2 overexpression plasmid, or miR-181a-5p overexpression lentivirus plus MEG2 overexpression plasmid (1 × 10^7^ cells per mouse, 5 mice per group). The mice were sacrificed 21 days after injection. The mouse gastric tumours were removed and weighed. Parts of the tissues were used for total RNA and protein extraction. The remaining parts were fixed in 4% paraformaldehyde for 24 h at 4 °C and used for immunocytochemistry and haematoxylin and eosin (H&E) staining. All procedures were approved by the Institutional Review Board of Nanjing University (Nanjing, China) and performed in accordance with the guidelines of the National Institutes of Health and the U.K. Animals (Scientific Procedures) Act (1986).

### Statistical analysis

All experiments were independently repeated at least three times. The data shown are the mean ± SEM. *P*-values of less than 0.05 were considered statistically significant using two-tailed Student’s t-test.

## Results

### MEG2 was downregulated in human gastric cancer

**Fig. 1:**
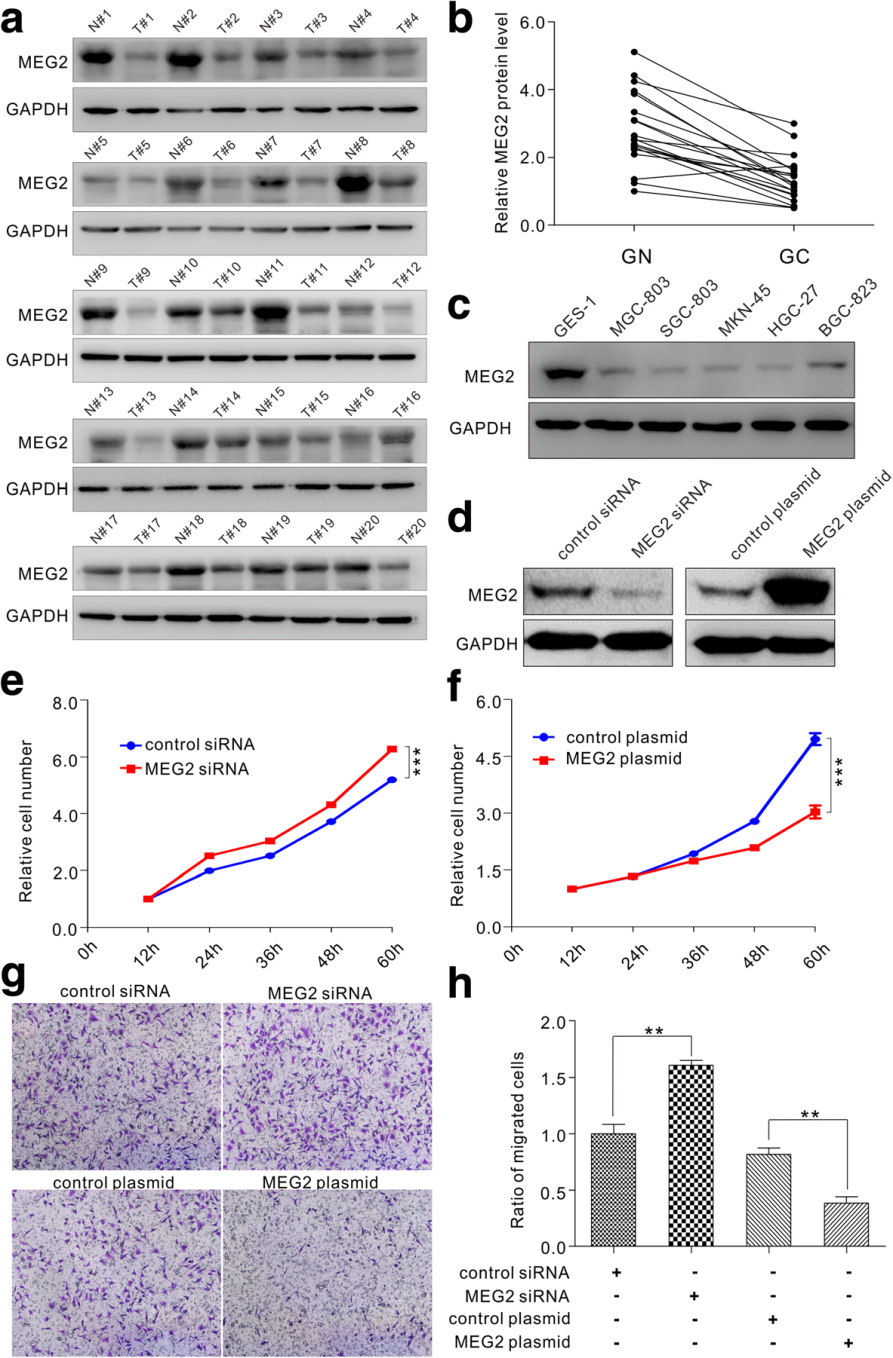
MEG2 is downregulated in human gastric cancer and functions as a tumour suppressor gene to suppress the proliferation and migration of gastric cancer cells. **a** and **b** Western blot analysis of MEG2 protein in 20 paired human gastric cancer specimens (GC) and normal adjacent gastric tissue (GN) samples. **a** representative image; **b** quantitative analysis. **c** Western blot analysis of MEG2 protein in 5 human GC cell lines and 1 human gastric epithelial cell line. **d** Western blot analysis of MEG2 protein in MGC803 cells transfected with MEG2 siRNA, control siRNA, MEG2 plasmid and control plasmid. **e** Cell proliferation assays were performed after the transfection of MGC803 cells with MEG2 siRNA and control siRNA in equal doses. **f** Cell proliferation assays were performed after the transfection with MEG2 plasmid and control plasmid in equal doses. **g** and **h** Transwell migration assays were performed after the transfection with MEG2 siRNA, control siRNA, MEG2 plasmid and control plasmid. **g** representative images; **h** quantitative analysis. * *P* < 0.05; ** *P* < 0.01; *** *P* < 0.001

To determine the level of MEG2 expression in human gastric cancer specimens, we measured MEG2 protein in 20 pairs of gastric cancer specimens and corresponding adjacent specimens. As shown in Fig. 1a and b, expression levels of MEG2 protein were strikingly lower in gastric cancer specimens compared to adjacent noncancerous specimens. In addition, we measured the expression levels of MEG2 protein in 5 human GC cell lines (MGC803, SGC7901, MKN-45, HGC-27 and BGC-823) and 1 human gastric epithelial cell line (GES-1). MEG2 protein was weakly present in 5 GC cell lines and highly present in GES-1 cell line (Fig. 1c and Additional file 3: Figure S2A). These results demonstrated that MEG2 was downregulated in human gastric cancer.

### MEG2 functioned as a tumour suppressor gene in gastric cancer cells

We then evaluated the biological function of MEG2 in gastric cancer. Previous studies have demonstrated that MEG2 is essential for suppressing proliferation and migration of breast cancer cells [5, 14]. Thus, CCK-8 and Transwell assays were performed to analyse the effect of MEG2 on the proliferation and migration of gastric cancer cells. MGC803 cells transfected with MEG2 siRNA showed an increase in cell proliferation and migration (Fig. 1e–h), while those transfected with the MEG2-overexpressing plasmid suppressed cell proliferation and migration (Fig. 1f–h). Efficient overexpression and knockdown of MEG2 expression in gastric cancer cells are shown in Fig. 1d, Additional file 3: Figures. S2B, S2C. These results suggested that MEG2 may function as a tumour suppressor gene and suppress proliferation and migration in gastric cancer cells.

### MEG2 was predicted as a target gene of miR-181a-5p

**Fig. 2:**
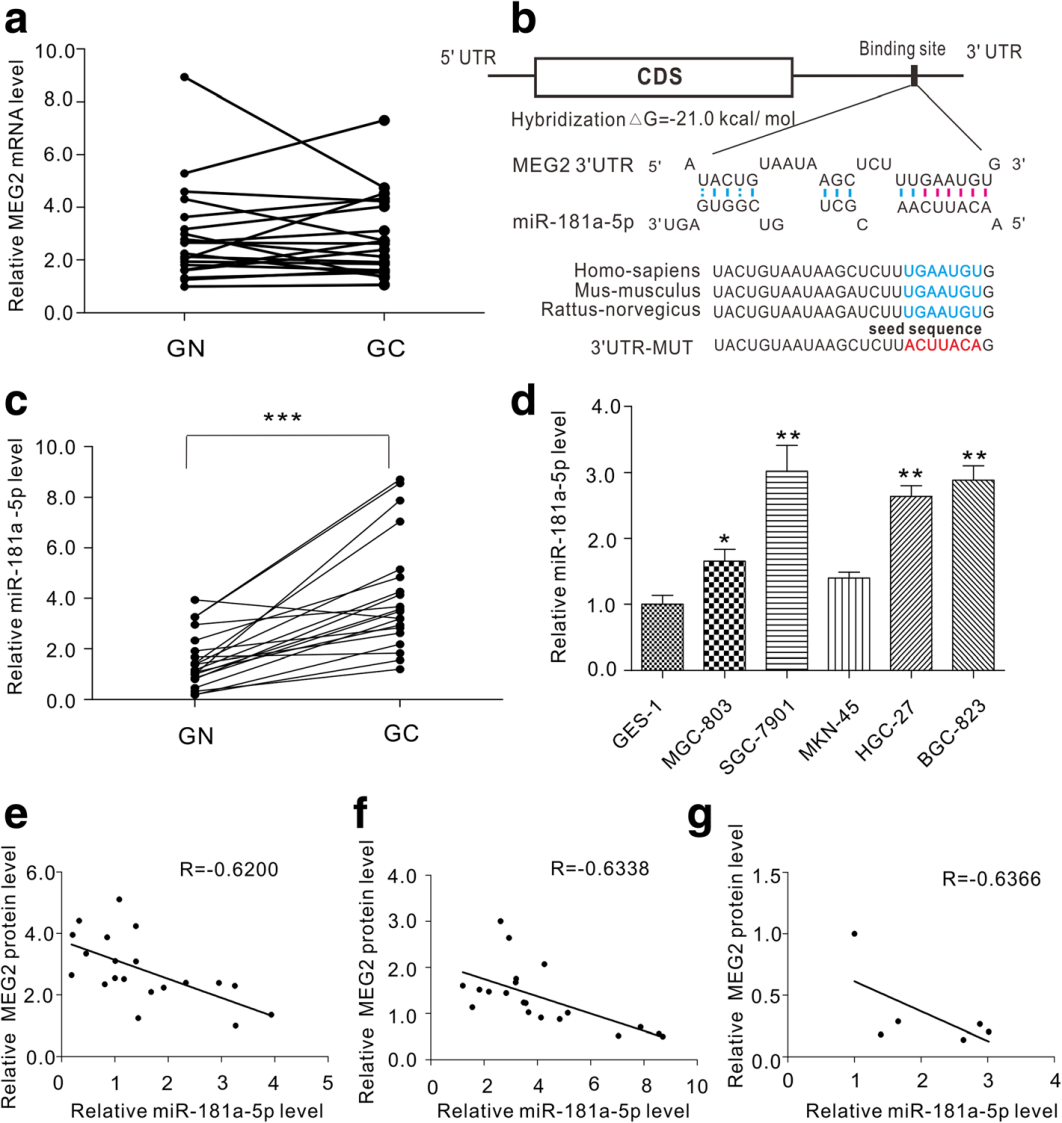
Prediction of MEG2 as a miR-181a-5p target. **a** Quantitative RT-PCR analysis of MEG2 mRNA expression levels in the same 20 pairs of GC and GN specimens. **b** Schematic description of the hypothetical duplex formed by the interaction between the MEG2 3′-UTR binding site (top) and miR-181a-5p (bottom). The calculated free energy value of the hybrid is indicated. The seed recognition sites are denoted in red. All nucleotides of the seed recognition sites are highly conserved in several species. **c **Quantitative RT-PCR analysis of miR-181a-5p expression levels in the same specimens. **d** Quantitative RT-PCR analysis of miR-181a-5p expression levels in the same gastric cell lines. **e** Pearson’s correlation scatter plot of the correlation between MEG2 protein and miR-181a-5p in GN specimens. **f** Pearson’s correlation scatter plot of the correlation between MEG2 protein and miR-181a-5p in GC specimens. **g** Pearson’s correlation scatter plot of the correlation between MEG2 protein and miR-181a-5p in 5 human GC cell lines and 1 human gastric epithelial cell line. *P < 0.05; **P < 0.01; ***P < 0.001

To further investigate the potential mechanism of downregulation of MEG2, we measured the mRNA levels of MEG2 in the same specimens pairs described above. We detected irregular alterations of MEG2 mRNA between the cancerous and adjacent normal specimens (Fig 2a). The inconsistency between MEG2 mRNA and protein in gastric cancer specimens suggested that a post-transcriptional mechanism is involved in the regulation of MEG2 protein in gastric cancer. We hypothesized that miRNAs, which represent an important way of regulating gene expression at the post-transcriptional level, suppress MEG2 expression levels in human gastric cancer specimens. To predict potential miRNAs that target MEG2, we scanned three computational algorithms (TargetScan20 [30], miRanda8 [31] and PicTar21 [30]). We chose miR-181a-5p for further experimentation because it was predicted as a regulator of MEG2 by all three software models. The predictive information between miR-181a-5p and the binding sites in the MEG2 3′-UTR is illustrated in Fig. 2b. We further analysed the minimum free energy value of the hybrid between miR-181a-5p and the binding site on the MEG2 3′-UTR. The minimum free energy value was −21.0 kcal/mol, which is within the range of genuine miRNA-target pairs. Additionally, there was perfect base-pairing between the seed region and the cognate target, which means that the miR-181a-5p binding sequence in the MEG2 3′-UTR is highly conserved among species (Fig. 2b).

### miR-181a-5p and MEG2 levels were inversely correlated in human gastric cancer

We further measured miR-181a-5p levels in the same 20 pairs of gastric cancer specimens and adjacent noncancerous specimens. As shown in Fig. 2c, the miR-181a-5p levels were higher in the gastric cancer specimens. We also directly illustrated the inverse correlation between miR-181a-5p and MEG2 protein by Pearson’s correlation scatter plots in adjacent noncancerous specimens (Fig. 2e) and GC specimens (Fig. 2f), respectively. Moreover, we examined the expression level of miR-181a-5p in 6 gastric cell lines mentioned before to confirm the inverse correlation between miR-181a-5p and MEG2 protein in vitro (Fig. 2d). As expected, MEG2 expression level was inversely correlated with miR-181a-5p in these gastirc cell lines (Fig. 2g). Based on the above findings, we determined MEG2 to be a likely target of miR-181a-5p.

### miR-181a-5p reduced MEG2 expression directly at the post-transcriptional level

**Fig. 3:**
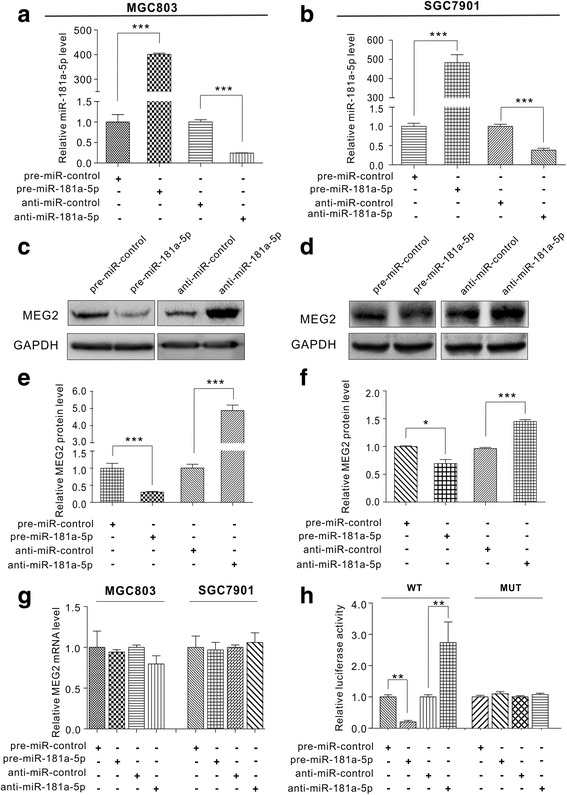
miR-181a-5p directly regulates MEG2 expression. **a** and **b** Quantitative RT-PCR analysis of miR-181a-5p levels in MGC803 and SGC7901 cells transfected with pre-miR-181a-5p, pre-miR-control, anti-miR-181a-5p or anti-miR-control in equal doses. **c**-**f** Western blot analysis of MEG2 protein in MGC803 and SGC7901 cells transfected with pre-miR-181a-5p, pre-miR-control, anti-miR-181a-5p or anti-miR-control. C and D: representative image; E and F: quantitative analysis. **g** Quantitative RT-PCR analysis of MEG2 mRNA levels in MGC803 and SGC7901 cells transfected with pre-miR-181a-5p, pre-miR-control, anti-miR-181a-5p or anti-miR-control in equal doses. **h** Direct binding of the MEG2 3′-UTR by miR-181a-5p. Firefly luciferase reporters containing the wild-type (WT) or mutant (MUT) form in the MEG2 3′-UTR were transfected into MGC803 cells with pre-miR-181a-5p, pre-miR-control, anti-miR-181a-5p or anti-miR-control. Luciferase assays were performed 24 h after transfection. Firefly luciferase values were normalized to β-galactosidase activity, and the results were plotted as relative luciferase activity. The luciferase activity in the control cells was set as 1. *P < 0.05; **P < 0.01; ***P < 0.001

To verify the inverse correlation between miR-181a-5p and MEG2, we used western blotting to evaluate MEG2 expression levels in human gastric cancer cells after miR-181a-5p overexpression or knockdown. miR-181a-5p was efficiently overexpressed or knocked down in MGC803 cells as shown in Fig. 3a. Subsequently, MEG2 expression levels significantly decreased upon miR-181a-5p overexpression and increased upon knockdown of miR-181a-5p in MGC803 cells (Fig. 3c and e). We also evaluated MEG2 mRNA expression levels after miR-181a-5p overexpression or knockdown (Fig. 3g). To confirm the robustness, we used another gastric cell line (SGC7901) to repeat the above experiments and observed consistent results (Fig. 3b–g).

To further determine whether miR-181a-5p regulated MEG2 expression by directly interacting with the binding site in the MEG2 3′-UTR, the target sequence of MEG2 3′-UTR was cloned into a luciferase reporter vector. The synthetic plasmid was co-transfected with pre-miR-181a-5p, anti-miR-181a-5p or scrambled negative control RNAs into MGC803 cells. As expected, miR-181a-5p overexpression led to an approximately 80% decrease in luciferase reporter activity, whereas miR-181a-5p inhibition led to a nearly three-fold increase in reporter activity (Fig. 3h). As a negative control, a mutant plasmid in which the miR-181a-5p binding site in the MEG2 3′-UTR was introduced by point mutations was engineered to completely disrupt the miR-181a-5p binding ability. The luciferase activity of the mutated reporter was not obviously affected by either miR-181a-5p overexpression or knockdown (Fig. 3h). Taken together, these results suggest that miR-181a-5p directly recognizes and binds to the 3′-UTR of the MEG2 transcript and inhibits MEG2 translation.

### MEG2 and miR-181a-5p had opposing effects on cell proliferation and migration in gastric cancer cells

We proposed that miR-181a-5p promoted the gastric oncogenic process by inhibiting MEG2 expression. Thus, we investigated the effect of miR-181a-5p on cell proliferation and migration using CCK-8 and Transwell assays. MGC803 cells transfected with pre-miR-181a-5p had significantly greater capabilities to proliferate and migrate, while miR-181a-5p inhibition showed the opposite effect (Additional file 4: Figure S3). Consequently, we found that miR-181a-5p had opposing effects on cell proliferation and migration in gastric cancer cells compared with MEG2 (Fig. 1e-h).

**Fig. 4:**
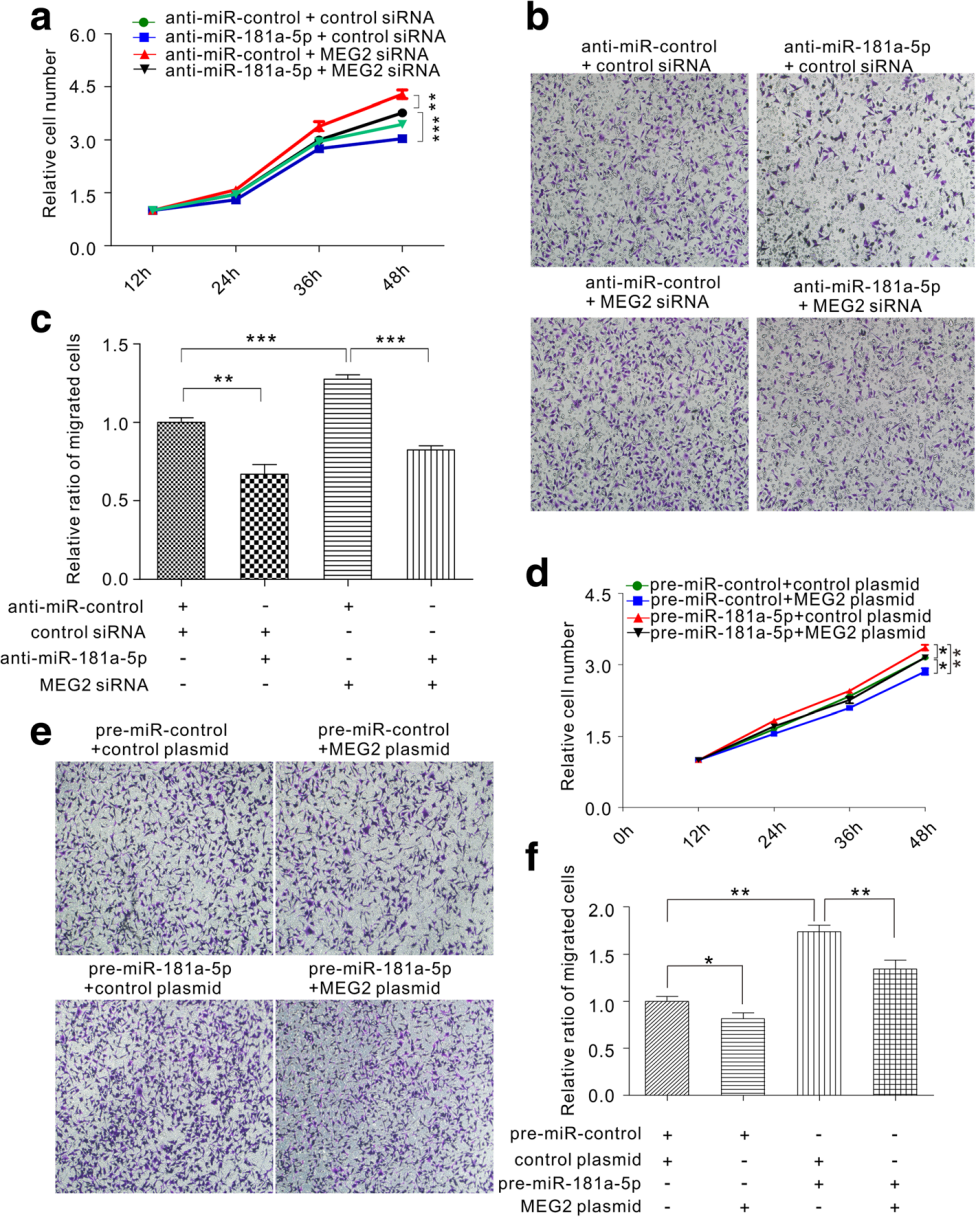
Effects of miR-181a-5p and MEG2 on the proliferation and migration of gastric cancer cells. **a** Cell proliferation assays were performed after the transfection with anti-miR-control plus control siRNA; anti-miR-181a-5p plus control siRNA; anti-miR-control plus MEG2 siRNA; or anti-miR-181a-5p plus MEG2 siRNA in equal doses. **b** and **c** Transwell analysis of MGC803 cells transfected with anti-miR-control plus control siRNA; anti-miR-control plus MEG2 siRNA; anti-miR-181a-5p plus control siRNA; or anti-miR-181a-5p plus MEG2 siRNA in equal doses. **b** representative image; **c** quantitative analysis. **d** Cell proliferation assays were performed after the transfection with pre-miR-control plus control plasmid; pre-miR-control plus MEG2 overexpression plasmid; pre-miR-181a-5p plus control plasmid; or pre-miR-181a-5p plus MEG2 overexpression plasmid in equal doses. **e** and **f** Transwell analysis of MGC803 cells transfected with pre-miR-control plus control plasmid; pre-miR-control plus MEG2 overexpression plasmid; pre-miR-181a-5p plus control plasmid; or pre-miR-181a-5p plus MEG2 overexpression plasmid in equal doses. **e** representative image; **f** quantitative analysis. *P < 0.05; **P < 0.01; ***P < 0.001

To determine whether miR-181a-5p modulated gastric cancer cells by directly targeting MEG2, we subsequently investigated the effect of miR-181a-5p-MEG2 on gastric cancer cell proliferation and migration. We co-transfected gastric cancer cells with the following synthetic RNAs: (1) anti-miR-control and control siRNA, (2) anti-miR-181a-5p and control siRNA (3) anti-miR-control and MEG2 siRNA, and (4) anti-miR-181a-5p and MEG2 siRNA. As expected, gastric cancer cells co-transfected with anti-miR-181a-5p and MEG2 siRNA showed significantly improved capabilities to proliferate (Fig. 4a) and migrate (Fig. 4b and c) compared to the cells transfected with anti-miR-181a-5p alone. These findings illustrated that the effect of anti-miR-181a-5p in inhibiting cellular proliferation and migration was efficiently restored by co-transfection of MEG2 siRNA. Conversely, we also co-transfected gastric cancer cells with the following synthetic RNAs/plasmids: (1) pre-miR-control and control plasmid, (2) pre-miR-control and MEG2 overexpression plasmid with full-length ORF lacking the miR-181a-5p-responsive 3′-UTR, (3) pre-miR-181a-5p and control plasmid, and (4) pre-miR-181a-5p and MEG2 overexpression plasmid. Consistent with previous results, gastric cancer cells co-transfected with pre-miR-181a-5p and MEG2 overexpression plasmid showed significantly lower capabilities to proliferate (Fig. 4d) and migrate (Fig. 4e and f) compared to the ones transfected with pre-miR-181a-5p alone. These findings indicate that overexpression of MEG2 rescues the MEG2 suppression caused by miR-181a-5p and attenuates miR-181a-5p–mediated carcinogenic effects, represented by enhanced cell proliferation and migration in gastric cancer cells. Taken together, we demonstrated that miR-181a-5p might promote cell proliferation and migration by inhibiting MEG2.

### miR-181a-5p promoted the growth of gastric cancer cells by inhibiting MEG2 in vivo

**Fig. 5:**
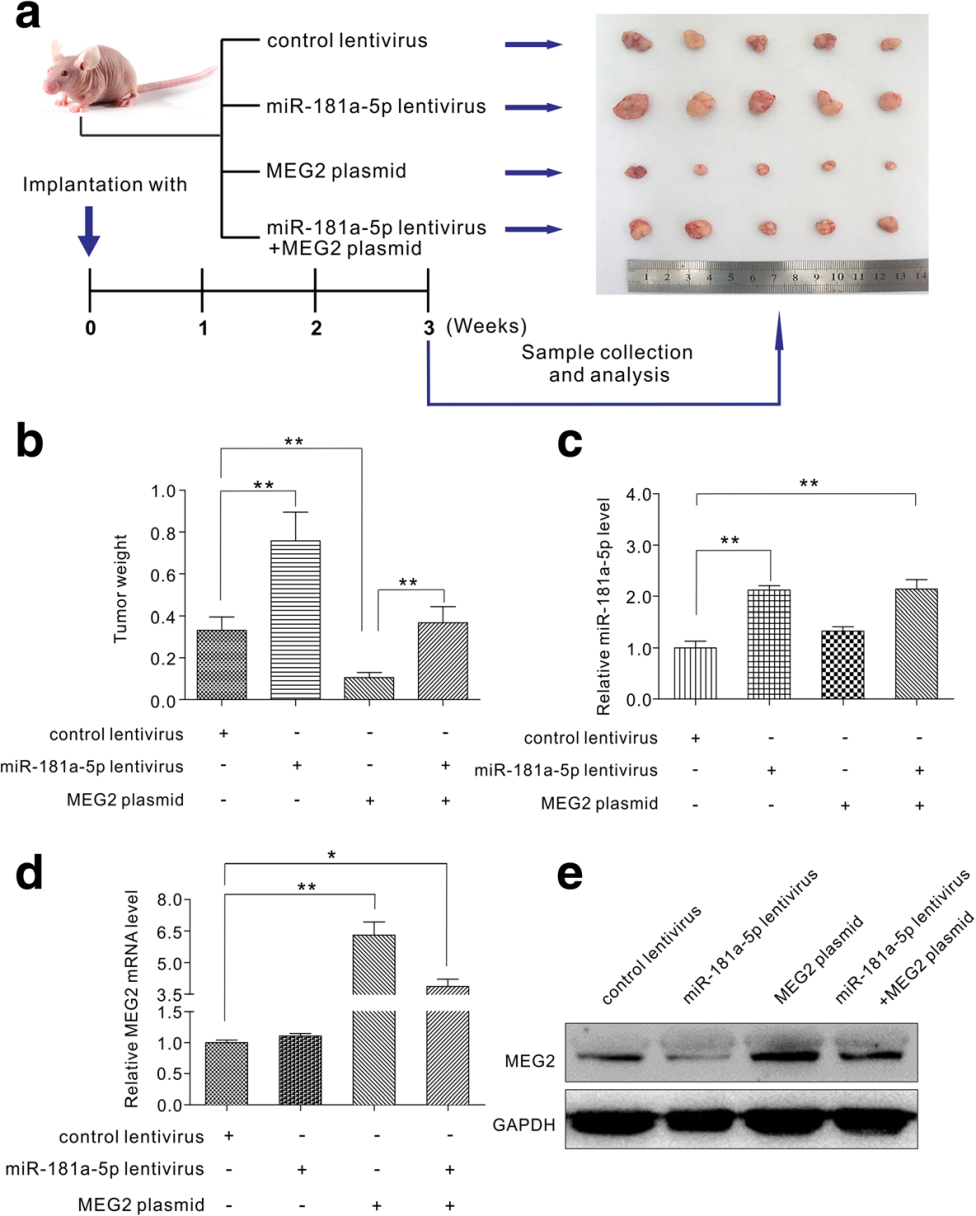
Effects of miR-181a-5p and MEG2 on the growth of gastric cancer xenografted tumours in vivo. **a** Flowchart of experimental layout and representative images of xenografted tumours from implanted mice. MGC803 cells were divided into four groups and were infected with control lentivirus, miR-181a-5p overexpression lentivirus, MEG2 overexpression plasmid, or miR-181a-5p overexpression lentivirus plus MEG2 overexpression plasmid. These differentially treated MGC803 cells were subcutaneously implanted into 5-week-old SCID mice (1 × 10^7^ cells per mouse and 5 mice per group). After 21 days, tumour growth was evaluated. **b** Quantitative analysis of xenografted tumour weights. **c** Quantitative RT-PCR analysis of miR-181a-5p expression levels in xenografted tumours. **d** Quantitative RT-PCR analysis of MEG2 mRNA expression levels in xenografted tumours. **e** Representative image of western blot analysis of MEG2 protein expression levels in xenografted tumours. * *P* < 0.05; ** *P* < 0.01; *** *P* < 0.001

To further confirm the above findings in vivo, we established gastric cancer xenografts in mice to observe the effects of miR-181a-5p and MEG2. MGC803 cells were infected with (1) control lentivirus, (2) miR-181a-5p overexpression lentivirus, (3) MEG2 overexpression plasmid, and (4) miR-181a-5p overexpression lentivirus together with MEG2 overexpression plasmid. Infected or transfected MGC803 cells were implanted into 5-week-old SCID mice. After three weeks of xenograft growth in vivo, we sacrificed the mice and assessed tumour growth. We compared the sizes of xenograft tumours among the groups and found that the group with miR-181a-5p-overexpression showed a significant increase in both parameters compared to the control group, whereas the MEG2-overexpressing group exhibited a dramatic decrease (Fig. 5a and b). In addition, MEG2 overexpression attenuated the promotion of miR-181a-5p (Fig. 5a and b), suggesting that miR-181a-5p promotes tumour growth by inhibiting MEG2. We subsequently extracted total RNA and protein from the tumours and analysed miR-181a-5p and MEG2 expression. As expected, the mRNA levels of MEG2 were higher in the tumour tissues from the MEG2-overexpressing group (Fig.5d). The miR-181a-5p expression levels of tumour tissues from the miR-181a-5p-overexpressing group was significantly higher compared to that from the control group (Fig. 5c). With regards to MEG2 protein levels, the miR-181a-5p-overexpressing group exhibited lower levels compared to the control group, whereas the MEG2-overexpressing group exhibited elevated levels (Fig. 5e and Additional file 5: Figure S4A). Additionally, compared to the group overexpressing MEG2 alone, tumours from both the miR-181a-5p and MEG2 overexpression groups showed significantly lower MEG2 levels (Fig. 5e and Additional file 5: Figure S4A). These results further confirmed that MEG2 overexpression rescues MEG2 suppression caused by miR-181a-5p in vivo. Moreover, H&E staining showed that miR-181a-5p lentivirus promotes cell mitosis while MEG2 plasmid downregulates cell mitosis. Similarly, both miR-181a-5p and MEG2 overexpression groups showed less cell mitosis compared to the one with miR-181a-5p overexpression alone (Additional file 5: Figure S4B). Immunocytochemistry with mouse monoclonal antibody against Ki-67 was used to assess the proliferative activity of the tumour cells. The cell proliferation rate was higher in the tumour tissues from the miR-181a-5p-overexpressing group and lower in the tumour tissues from the MEG2-overexpressing group (Additional file 5: Figure S4B). Taken together, we demonstrated that MEG2 overexpression attenuated the promotive effect of tumour growth caused by miR-181a-5p overexpression. Thus, the in vitro and in vivo findings congruently validated the regulation of miR-181a-5p on the tumour suppressor activity of MEG2 in gastric tumourigenesis.

## Discussion

Surgery is a major treatment for GC, augmented with chemotherapy and/or radiotherapy. However, many advanced GC patients have a poor prognosis. Recently, clinicians have systemically measured the activity of potential genetic targets in tumours for GC therapy. Although trastuzumab has become a first-line treatment option for HER-2-positive advanced GC, there is no internationally accepted standard of cure, and survival of most advanced GC patients remains poor [32, 33]. Consequently, more effective therapeutic approaches to GC are much needed.

Multiple genetic alterations have been proved to be involved in gastric tumourigenesis. Oncogenes such as *HER-2* have been found to be overexpressed in this type of cancer [34]. In contrast, many tumour suppressor genes (such as *PTEN* and *P53*) are inactivated or downregulated [35]. The reversible regulation of signalling proteins by phosphorylating their certain tyrosine residues is one of the most common mechanisms in regulation of cellular signal transduction. PTPs are involved in the regulation of many cellular functions, such as proliferation and migration, by dephosphorylating relevant signalling proteins [36]. It is well known that dysfunction of PTPs is associated with various cancers and most of them function as tumour suppressors [36]. As one of the non-receptor PTPs, MEG2 was reported to dephosphorylates EGFR and HER-2 specifically, thereby indirectly inhibiting the activation of EGF-induced STAT3 and negatively regulating cell proliferation and migration [13]. In addition, Su et al. also found that MEG2 directly catalyzes dephosphorylation of STAT3 activated by v-Src at residue Try705 [14]. Since EGFR, HER-2 and STAT3 were known as important proto-oncogenes in gastric tumourigenesis, MEG2 might be an effective tumour suppressor in GC [33, 35, 37]. In this study, we demonstrated that MEG2 protein was significantly lower in both GC specimens and GC cell lines, compared to normal adjacent gastric mucosa and normal gastric cell lines. Furthermore, our functional experiment showed that MEG2 efficiently inhibited proliferation and migration in vitro and markedly attenuated tumour growth in vivo. These results confirmed that MEG2 functions as a tumour suppressor gene in GC.

In this study, the alteration trend of MEG2 mRNA between GC specimens and normal adjacent gastric mucosa was not consisted with that of MEG2 protein. This phenomenon inspired us that the expression of MEG2 protein might be regulated by a post-transcriptional mechanism. As a crucial post-transcriptional regulator, miRNA regulates expression of target genes by the imperfect complementary binding with their mRNAs [17]. Previous studies validated that several miRNAs (e.g., miR-24 and miR-96) were involved in regulation of MEG2 in breast cancer [5, 38]. Since the detail mechanism through which MEG2 was downregulated in GC remained unclear, we screened miRNAs that potentially target MEG2 by bioinformatics analyses and identified miR-181a-5p as a candidate. Subsequently, we showed that expression level of miR-181a-5p was inversely correlated with MEG2 protein level in both GC specimens and gastric cell lines. Furthermore, MEG2 was validated as a direct target of miR-181a-5p in two gastric cancer cells by knocking down and overexpressing miR-181a-5p. Thus, modulation of MEG2 by miR-181a-5p may explain, at least in part, why the upregulation of miR-181a-5p can promote cell proliferation and migration and tumor growth in gastric cancer. However, conflicting reports regarding the role of miR-181a-5p in suppressing or promoting tumorigenesis in different cancer types have left unanswered questions. Studies showed that miR-181a-5p was downregulated in several cancers, such as oral squamous cell carcinoma, glioma and leukemia [39–41]. Even in GC, also it was suggested that miR-181a-5p inhibited migration and proliferation of HGC-27 cells by targeting Prox1 [42]. Based on these findings, miR-181a-5p was supposed to be a tumour suppressor. In contrast, a number of studies showed that miR-181a-5p was upregulated and acted as an oncomiR in gastric carcinogenesis by suppressing RASSF6, ATM or KLF6 expression [29, 43–45]. In accordance with these findings, we showed that miR-181a-5p promoted proliferation and migration of GC cells and enhanced tumour growth in vivo. In addition, we observed that restoration of MEG2 expression attenuated the effects of miR-181a-5p both in vitro and in vivo. Thus, the role of miR-181a-5p may be tumour-type specific and miR-181a-5p is more likely to be an oncogene in GC. Nevertheless, the underlying molecular mechanisms through which miR-181a-5p is involved in the development and progression of different cancers remain to be fully elucidated. Under different circumstances, miR-181a-5p may exert different functions.

Dysregulation of miRNAs played an important role in gastric tumourigenesis [19, 20]. Correction of cellular miRNA levels may emerge as a potential therapeutic strategy [46, 47]. Overexpressed miRNAs can be silenced using antagomirs [47]. Indeed, some scientists have already established the potential usefulness of miRNAs as therapeutic molecules against cancers, including the prevention of metastasis formation by silencing of miR-10b [47]. In our study, MEG2 was downregulated in GC and was suppressed by oncomiR miR-181a-5p. Transfection with anti-miR-181a-5p displayed an anti-tumour effect by increasing MEG2 expression both in GC cells and in xenografted mice. Thus, it is quite possible that treatment GC with miR-181a-5p antagomir may be a promising strategy for GC patients showing upregulation of miR-181a-5p. Further effort is needed to characterize the feasibility of targeting miR-181a-5p in GC therapy and develop cost-effective and simplified manipulation methods.

## Conclusions

In summary, this study provided new insight into the role of MEG2 in gastric cancer. We demonstrated that MEG2 is downregulated in gastric cancer as a result of the upregulation of miR-181a-5p. Consequently, this regulation is able to promote gastric cancer cell proliferation and migration in vitro and enhance tumour growth in vivo*.* These results suggest that MEG2 is a tumour suppressor gene that is negatively regulated by miR-181a-5p in human gastric cancer and might serve as a potential new target for future gastric cancer therapy.

## Additional files

Additional file 1: Table S1. Patients’ Characteristics. (DOCX 20 kb)

Additional file 2: FigureS1. Establishment of stably infected MGC803 cells. **a** The detail construct of miR-181a-5p overexpression lentivirus plasmid. **b** The representative fluorescence image of stably infected MGC803 cells. (TIFF 1047 kb)

Additional file 3: Figure S2. Expression of MEG2 protein in six gastric cell lines and efficiency of MEG2 knockdown and overexpression in GC cells. **a** Quantitative analysis of western blots of MEG2 protein in six gastric cell lines. **b** Quantitative RT-PCR analysis of MEG2 mRNA levels in MGC803 cells treated with MEG2 siRNA, scrambled control siRNA, MEG2 plasmid and control plasmid in equal doses. **c** Quantitative analysis of western blots of MEG2 protein in MGC803 cells treated with MEG2 siRNA, scrambled control siRNA, MEG2 plasmid and control plasmid in equal doses. *** *P* < 0.001; ** *P* < 0.01. (TIFF 101 kb)

Additional file 4: Figure S3. Effects of miR-181a-5p on the proliferation and migration of gastric cancer cells. (A and B) Cell proliferation assays were performed after the transfection of MGC803 cells with pre-miR-181a-5p, pre-miR-control, anti-miR-181a-5p or anti-miR-control in equal doses. (C and D) Transwell analysis of MGC803 cells transfected with pre-miR-181a-5p, pre-miR-control, anti-miR-181a-5p or anti-miR-control in equal doses. C: representative image; D: quantitative analysis. *** *P* < 0.001. (TIFF 2028 kb)

Additional file 5: Figure S4. Effects of MEG2 and miR-181a-5p on the growth of gastric cancer xenografted tumours in vivo. **a** Quantitative analysis of western blot analysis of MEG2 protein expression levels in xenografted tumours. **b** H&E and immunohistochemical staining for Ki-67 in xenografted tumours. ** *P* < 0.01. (TIFF 1211 kb)

## Abbreviations

3′-UTR: 3′ untranslated region
CCK-8: Cell Counting Kit-8
FBS: Fetal bovine serum
GC: Gastric cancer
H&E: Hematoxylin and eosin
MEG2: Protein-tyrosine phosphatase MEG2
miRNA: microRNA
ORF: Open reading frame
RT-PCR: Reverse transcription polymerase chain reaction
siRNA: small interfering RNA
β-gal: β-galactosidase
