# Supplementary material for: MEG2 is regulated by miR-181a-5p and functions as a tumour suppressor gene to suppress the proliferation and migration of gastric cancer cells
Source: Mol Cancer. 2017 Jul 26;16:133. doi: 10.1186/s12943-017-0695-7 (PMC5530520; doi:10.1186/s12943-017-0695-7)
Supplement: Supplementary file 1 — Patients’ Characteristics. (DOCX 20 kb) [file 12943_2017_695_MOESM1_ESM.docx]

**Additional file 1: Table S1.** Patients’ Characteristics

| Case No. | Age（years） | Gender | TNM Stage | Clinical History |
| --- | --- | --- | --- | --- |
| #1 | 63 | male | ⅠB | Gastric Cancer |
| #2 | 58 | male | ⅡB | Gastric Cancer |
| #3 | 65 | male | ⅡB | Gastric Cancer |
| #4 | 64 | male | ⅢA | Gastric Cancer |
| #5 | 50 | Female | ⅢC | Gastric Cancer |
| #6 | 56 | male | ⅡB | Gastric Cancer |
| #7 | 62 | male | ⅢB | Gastric Cancer |
| #8 | 63 | Female | ⅠB | Gastric Cancer |
| #9 | 71 | Female | ⅢC | Gastric Cancer |
| #10 | 50 | Female | ⅢB | Gastric Cancer |
| #11 | 65 | male | ⅢB | Gastric Cancer |
| #12 | 52 | Female | ⅠA | Gastric Cancer |
| #13 | 71 | male | ⅢC | Gastric Cancer |
| #14 | 60 | male | ⅠB | Gastric Cancer |
| #15 | 47 | Female | ⅢA | Gastric Cancer |
| #16 | 51 | male | ⅢC | Gastric Cancer |
| #17 | 56 | Female | ⅢB | Gastric Cancer |
| #18 | 70 | male | ⅢC | Gastric Cancer |
| #19 | 64 | male | ⅢB | Gastric Cancer |
| #20 | 79 | male | ⅡA | Gastric Cancer |
