# Supplementary figures and images for: MEG2 is regulated by miR-181a-5p and functions as a tumour suppressor gene to suppress the proliferation and migration of gastric cancer cells
Source: Mol Cancer. 2017 Jul 26;16:133. doi: 10.1186/s12943-017-0695-7 (PMC5530520; doi:10.1186/s12943-017-0695-7)

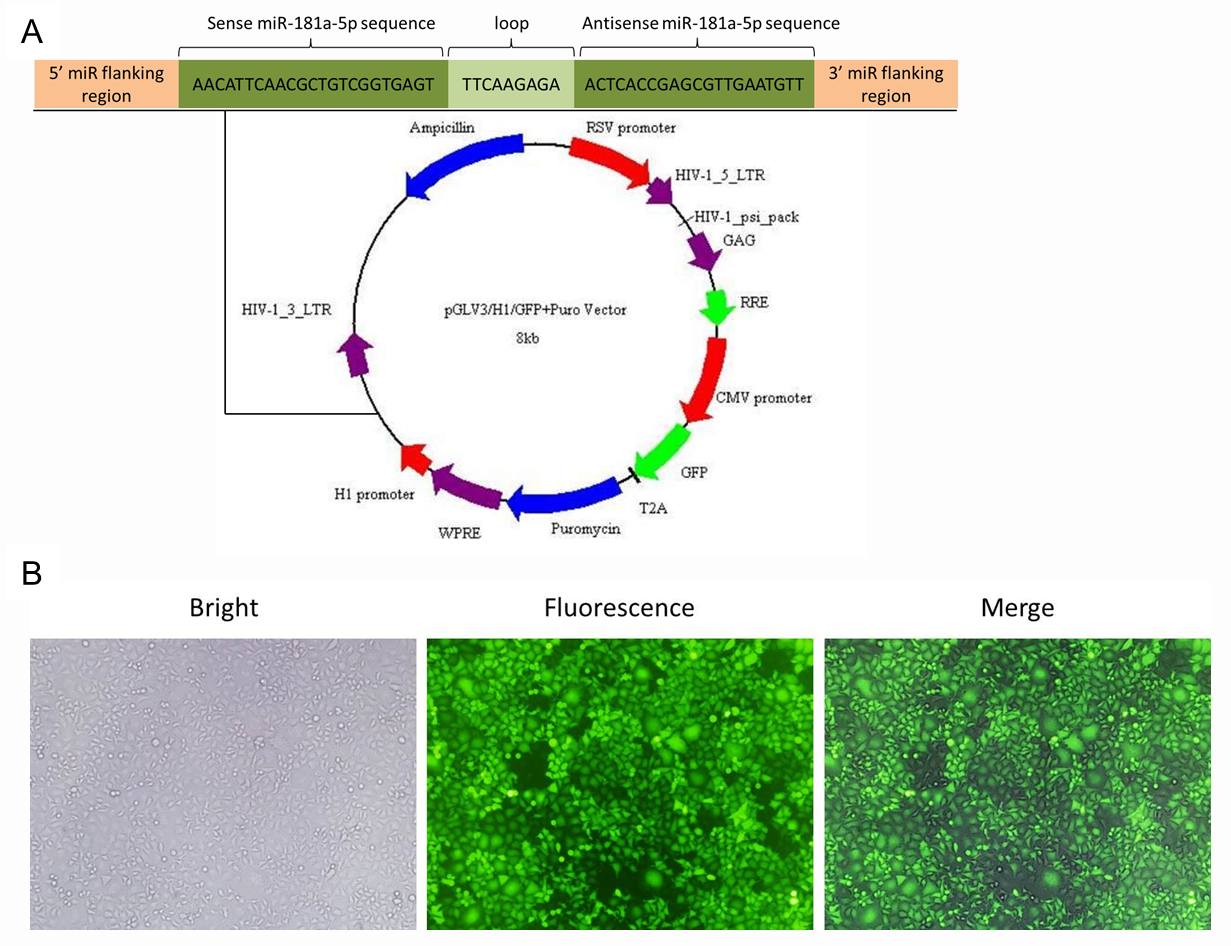

Supplement: Supplementary file 2 — Establishment of stably infected MGC803 cells. a The detail construct of miR-181a-5p overexpression lentivirus plasmid. b The representative fluorescence image of stably infected MGC803 cells. (TIFF 1047 kb) [file 12943_2017_695_MOESM2_ESM.tif]

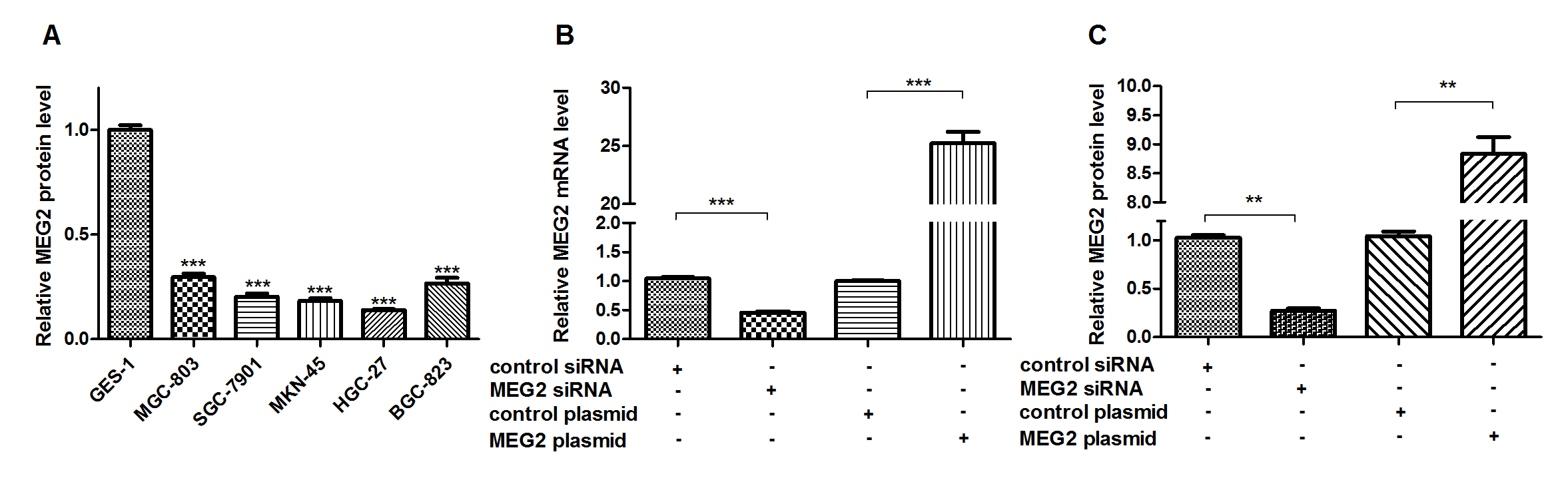

Supplement: Supplementary file 3 — Expression of MEG2 protein in six gastric cell lines and efficiency of MEG2 knockdown and overexpression in GC cells. a Quantitative analysis of western blots of MEG2 protein in six gastric cell lines. b Quantitative RT-PCR analysis of MEG2 mRNA levels in MGC803 cells treated with MEG2 siRNA, scrambled control siRNA, MEG2 plasmid and control plasmid in equal doses. c Quantitative analysis of western blots of MEG2 protein in MGC803 cells treated with MEG2 siRNA, scrambled control siRNA, MEG2 plasmid and control plasmid in equal doses. *** P < 0.001; ** P < 0.01. (TIFF 101 kb) [file 12943_2017_695_MOESM3_ESM.tif]

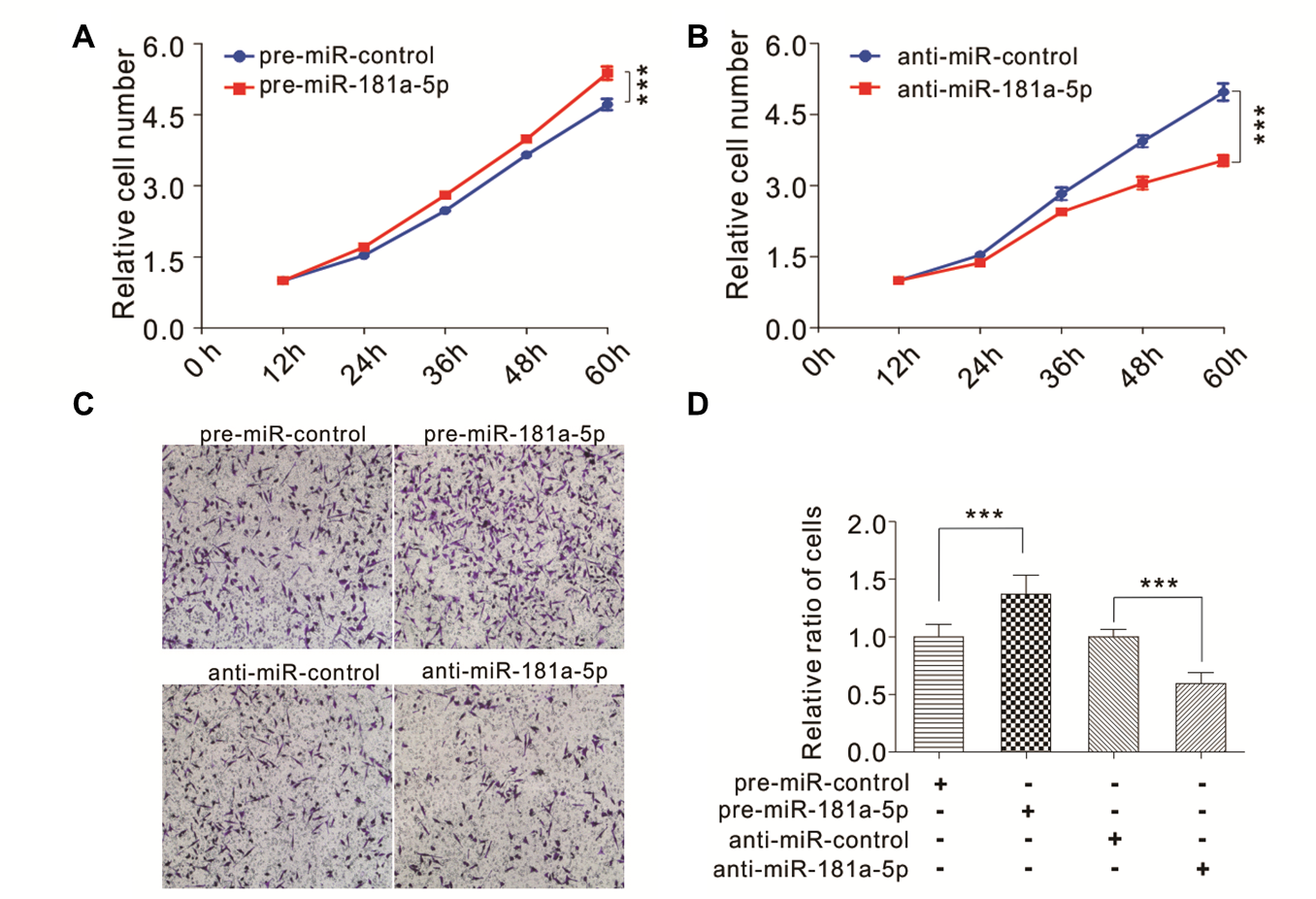

Supplement: Supplementary file 4 — Effects of miR-181a-5p on the proliferation and migration of gastric cancer cells. (A and B) Cell proliferation assays were performed after the transfection of MGC803 cells with pre-miR-181a-5p, pre-miR-control, anti-miR-181a-5p or anti-miR-control in equal doses. (C and D) Transwell analysis of MGC803 cells transfected with pre-miR-181a-5p, pre-miR-control, anti-miR-181a-5p or anti-miR-control in equal doses. C: representative image; D: quantitative analysis. *** P < 0.001. (TIFF 2028 kb) [file 12943_2017_695_MOESM4_ESM.tif]

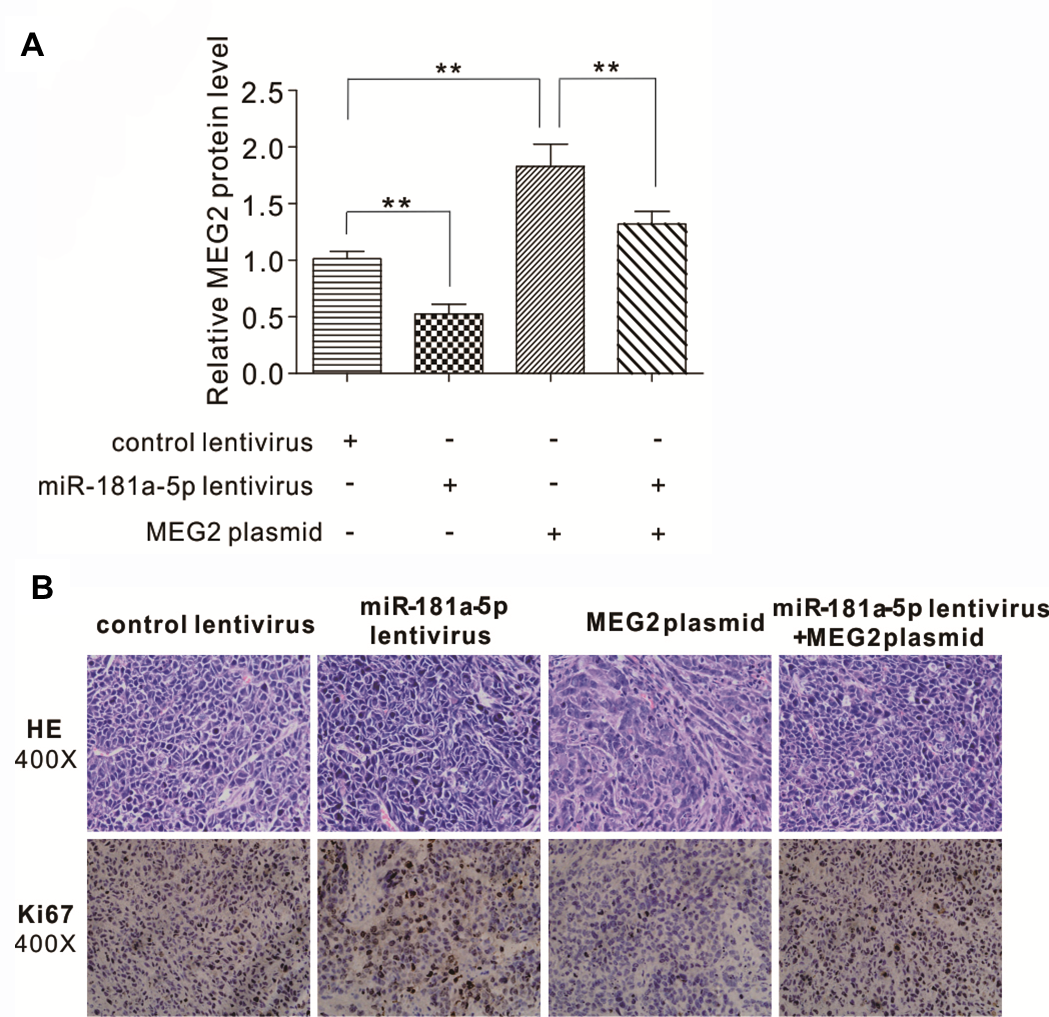

Supplement: Supplementary file 5 — Effects of MEG2 and miR-181a-5p on the growth of gastric cancer xenografted tumours in vivo. a Quantitative analysis of western blot analysis of MEG2 protein expression levels in xenografted tumours. b H&E and immunohistochemical staining for Ki-67 in xenografted tumours. ** P < 0.01. (TIFF 1211 kb) [file 12943_2017_695_MOESM5_ESM.tif]
